# Infantile Tremor Syndrome With Movement Disorder: Current Perspective

**DOI:** 10.7759/cureus.33091

**Published:** 2022-12-29

**Authors:** Sudesh Kumar, Surendra Sah, Prajna Ray, Piyali Bhattacharya, Nandita Chatterjee

**Affiliations:** 1 Pediatrics, Mata Gujri Memorial Medical College, Kishanganj, IND

**Keywords:** vitamin b12 deficiency, pallor, tremor, macrocytic anemia, breastfed

## Abstract

Although infantile tremor syndrome is considered a rare entity, we present a typical case of this disorder. This case reinforces the association of infantile tremor syndrome with exclusive breastfeeding in infants and the absence of proper complementary feeding. A nine-month-old, irritable, listless, exclusively breastfed female presented with grade 2 malnutrition, tremors, hyperpigmentation, scarce scalp hair, and delayed developmental milestones. Laboratory investigations revealed macrocytic anemia and a low serum vitamin B12 value of 205 pg/dL. Cerebral and mild cerebellar atrophy were noted on the MRI brain scan. Accordingly, the patient was diagnosed with infantile tremor syndrome and treated with vitamin B12 and nutrient supplementation with zinc, magnesium, folic acid, and iron. The tremors improved and the child became responsive and interested in her surroundings. It is essential to recognize this condition at the earliest and initiate treatment. Basic interventions such as the promotion of proper nutrition, timely introduction of complementary feeding, and weaning practices are key factors in decreasing the incidence of this condition.

## Introduction

Infantile tremor syndrome is a clinical entity marked by psychomotor delay or regression; coarse tremors of the head, tongue, and extremities; pallor; and pigmentary changes of hair and skin [1,2]. It is mostly found in children from India, Southeast Asia, and African countries. It accounts for about 0.2% of hospital admissions in the pediatric age group in India, with this figure showing a decreasing trend with time. Although rare, it is a concerning clinical condition reported in the age group of five months to three years due to its florid and serious presentation consisting of developmental changes, severe pallor, conspicuous pigmentary disturbance of hair and skin, and abnormal movement-like tremor [3]. The condition primarily seems to affect exclusively breastfed infants of vegetarian mothers [4]. However, the etiology is yet to be determined exhaustively, with research data mostly favoring nutritional deficiencies such as vitamin B12, iron, zinc, and magnesium [1,4]. The neurological symptoms can be explained by the low cerebrospinal fluid (CSF) levels of vitamin B12 and its transport protein transcobalamin-2. The diminishing incidence rate can be attributed to improvement in nutritional status and increased awareness about appropriate and timely weaning practices [5].

In this case report, we present a classical case of infantile tremor syndrome, highlighting the importance of proper nutrition and feeding practices in alleviating the manifestations of this rare but serious condition.

## Case presentation

**Figure 1:**
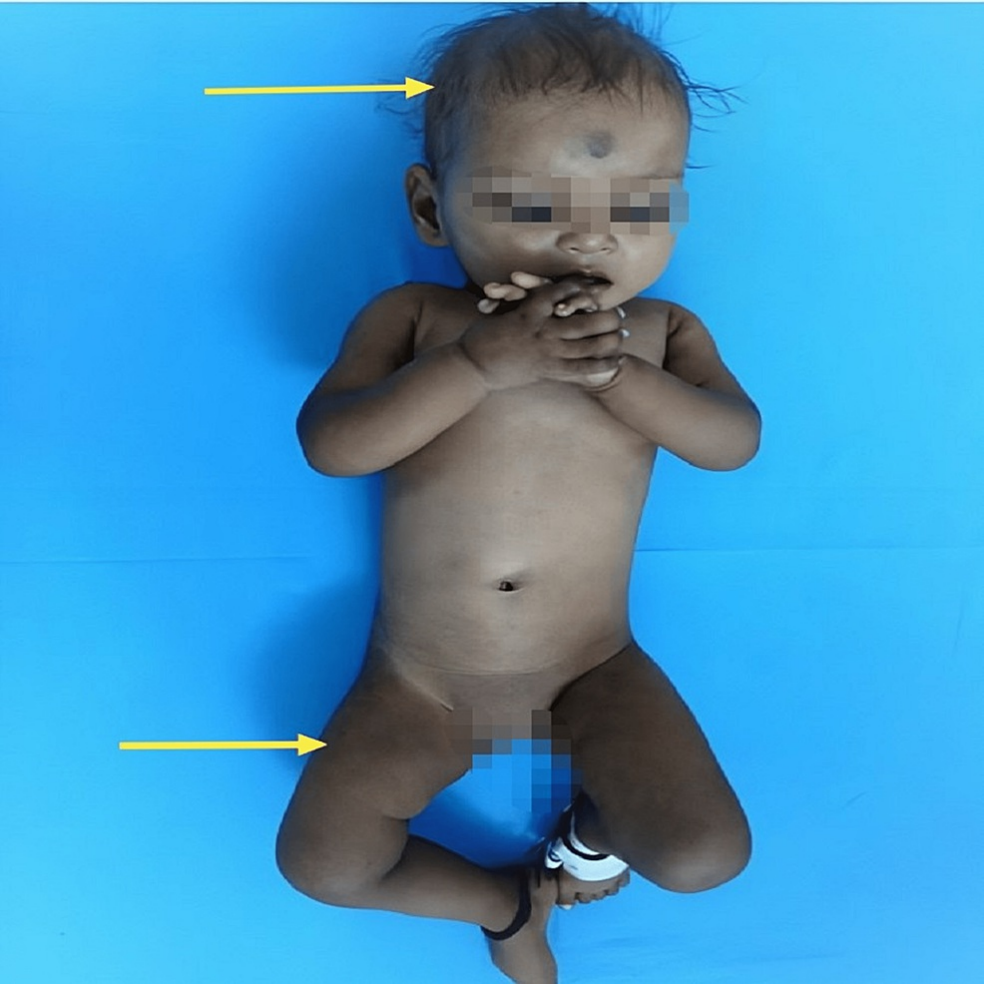
Sparse hair, hyperpigmented knuckles, and reticular pigmentation of the lower limbs.

A nine-month-old female child was brought to the outpatient department by her parents with the chief complaints of nodding of the head and tremors involving the distal part of all four extremities. They also noted that the child was unable to hold her neck unlike other children of the same age group. The abnormal movement began at the age of seven months (past two months), occurring only when the child was awake. There was no history of trauma, loss of consciousness, or any associated fever. The girl was born of a non-consanguineous marriage of a vegetarian, Hindu couple and was delivered via vaginal delivery at a government hospital. Her birth weight was 2.5 kg. There was no history of birth asphyxia, neonatal jaundice, fever, or any hospital admission. At present, the child’s weight was 5 kg, length was 64 cm, head circumference was 41 cm, and mid-upper arm circumference was 11 cm, which denoted that the child was suffering from grade 2 malnutrition (moderate) according to the Indian Academy of Paediatrics (IAP). Developmentally, the child could turn from a supine to a prone position and vice versa, attempt mouthing, grasp objects with fingers and thumb, and laugh aloud. The developmental quotient for gross motor was 66%, fine motor was 88.9%, adaptive was 55.5%, and language was 44.4%, suggestive of developmental delay. The child was exclusively breastfed till six months of age without the introduction of proper complementary feeding yet. Her diet mostly consisted of the breast milk of a vegetarian mother. The child was immunized according to the national immunization schedule. On examination, the child was plump and appeared irritable and apathetic. The scalp hair was sparse, coarse, and hypopigmented. Severe pallor was noted along with hyperpigmentation of knuckles and reticular appearance mainly in the extremities (Figure 1).

The complete blood count revealed severe anemia with leucopenia. The serum ferritin level was normal, and vitamin B12 levels of both the baby and the mother were in the lower normal range (Table 1).

**Table 1:** Laboratory investigations of the patient and the mother.

Investigations	Patient (reference values)	Mother
Red blood cell count	1.64 (3.5–5.2 × 10^6^/µL)	4.4 (4.2–6 × 10^6^/µL)
Hemoglobin	5 (12–16 g/dL)	12.7 (12–16 g/dL)
Mean corpuscular volume	109.5 (80–100 fL)	92 (787–100 fL)
Mean corpuscular hemoglobin	30.6 (27–34 pg)	30 (27–32 pg)
Mean corpuscular hemoglobin concentration	27.9 (31–37 g/dL)	32.8 (31–37 g/dL)
Total leucocyte count	4.78 (4,000–12,000/µL)	5.36 (4,000–11,000/µL)
Neutrophil, lymphocyte	8% (50–70%), 87% (20–60%)	
Serum ferritin	244 (30–400 ng/mL)	
Serum B12 level	205 (197–771 pg/mL)	207
Peripheral smear	Predominantly macrocyte and hypersegmented neutrophil	Predominantly normocytic normochromic red blood cells

**Figure 2:**
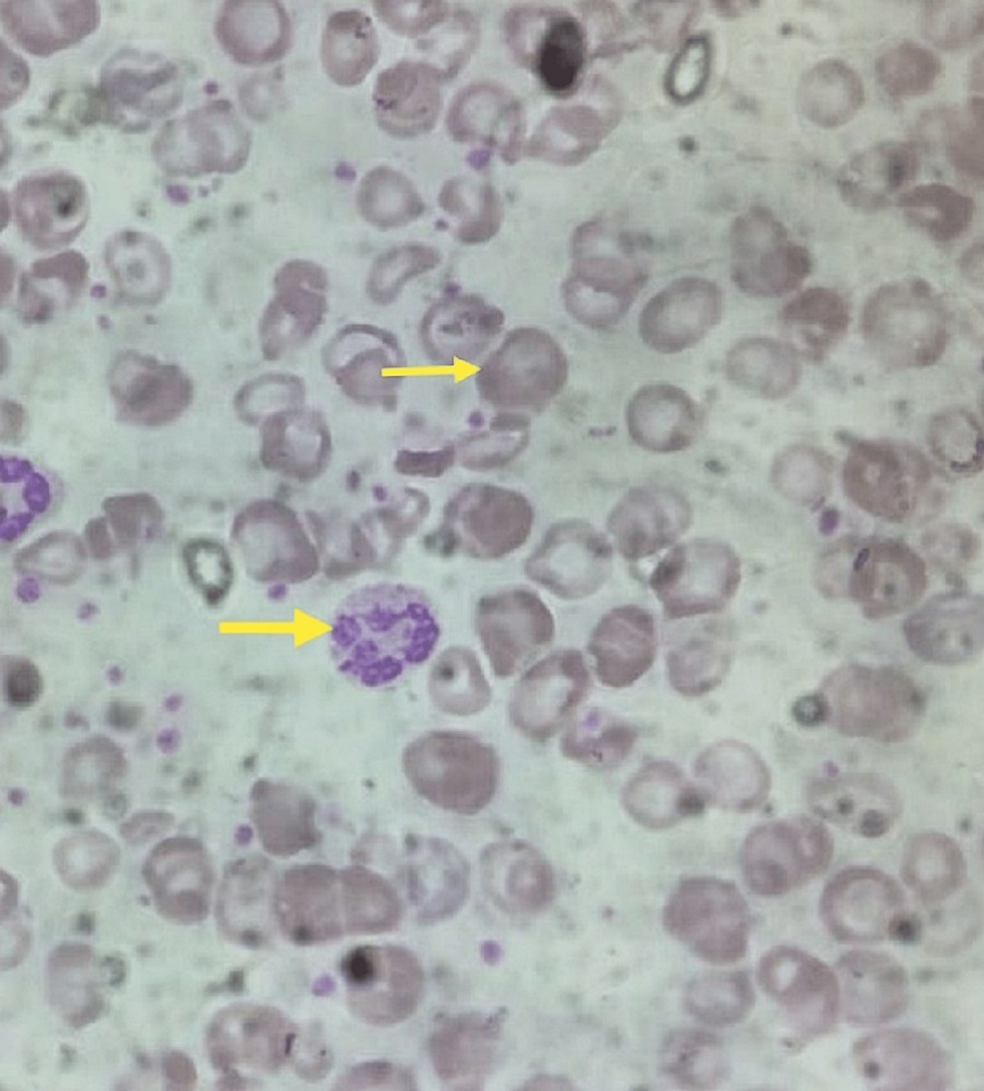
Peripheral blood smear showing macrocytes and hypersegmented neutrophils.

The peripheral blood smear was suggestive of macrocytic anemia with hypersegmented neutrophils (Figure 2).

**Figure 3:**
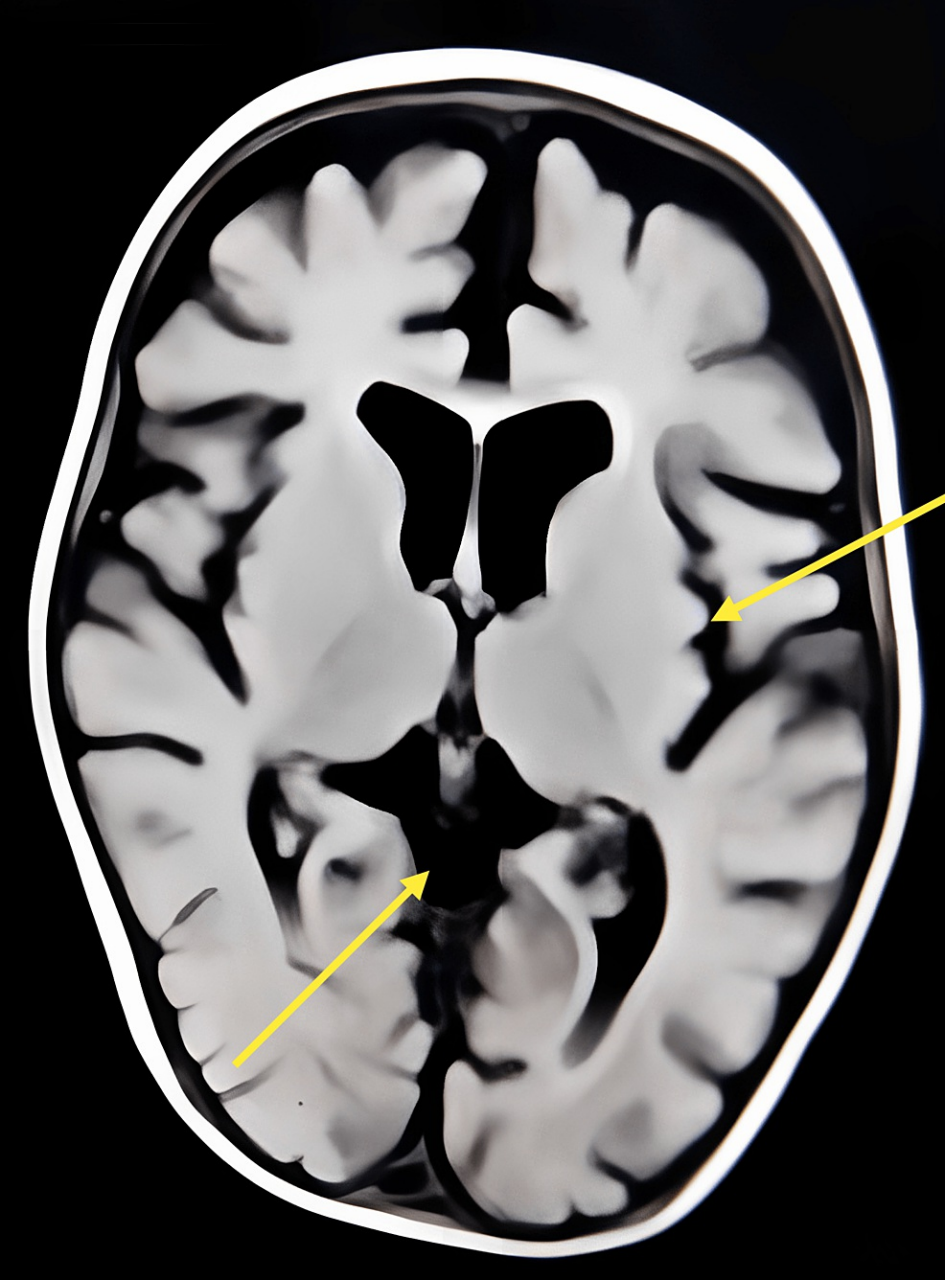
MRI showing cerebral atrophy and ventricular prominence.

MRI of the brain showed cerebral atrophy with prominent lateral ventricles, thinning of the corpus callosum, and mild cerebellar atrophy (Figure 3).

In the absence of any significant perinatal event, the vegetarian dietary habit of the mother, lack of complementary feeding, and investigation findings, a diagnosis of infantile tremor syndrome due to vitamin B12 deficiency with grade 2 malnutrition was made. Accordingly, the child was treated with an injection of vitamin B12 (1 mg intramuscular daily for two weeks, then weekly for four weeks) and rest as per the treatment guidelines of severe acute malnutrition (vitamin, folic acid, calcium, zinc, magnesium, and iron supplementation) and diet according to calorie requirements as per age. The mother was counseled about weaning off the baby and starting complementary feeding. The child started to take interest in the surroundings by day four of treatment with a cheerful disposition. The tremors also showed improvement by day five, and the hyperpigmentation started to fade by day seven.

## Discussion

In the majority of cases, infantile tremor syndrome classically presents with pallor; regression or delay of developmental milestones; hyperpigmentation of the skin; sparse, hypopigmented scalp hairs; and tremors [4]. Other features may include a plump appearance and apathy [3]. This disorder is often accompanied by nutrient or vitamin deficiencies [1]. The tremors are often acute in onset [4,5]; coarse in nature; involve the distal limbs, head, face, and tongue [4,5]; intermittent initially; precipitated by infection, stress, and external stimuli [4,5]; disappear during sleep; and are usually self-limiting [1]. Developmentally, delay or regression is noted around four to six months of age [4]. Variable degrees of malnutrition and vitamin deficiency are also found to be associated [6]. Our patient presented with typical features of tremors, pallor, developmental delay, skin pigmentation, apathetic attitude, scarce hairs at one year of age, and grade 2 malnutrition (according to the IAP). This syndrome is mostly found in children that are on prolonged exclusive breastfeeding [1], with mothers consuming only vegetables, and a lack of introduction of timely complementary feeding [6]. Our patient also had a similar background with feeding only on the breast milk of a vegetarian mother even at one year of age.

The hematological picture is that of anemia with the red blood cell morphology mostly being macrocytic or dimorphic [3,4]. The serum vitamin B12 level is recorded to be low in most cases [4,6]. Although serum B12 level was normal in our case, other features of vitamin B12 deficiency, such as megaloblastic anemia, were present. Some studies have also reported normal serum vitamin B12 levels [4]. Sometimes, the mother suffers from vitamin B12 deficiency resulting in low vitamin B12 levels in the breast milk [6]. The neuroimaging finding in these cases is cortical atrophy with involvement of the subarachnoid space [4,6], ventricular prominence, and myelinolysis of the pontine region [3].

The etiology of infantile tremor syndrome is still a matter of deliberation and research. Various causes such as infections, degenerative processes, and nutritional deficiencies such as iron, magnesium, and zinc have been proposed but not confirmed with certainty [1,4,6]. The most accountable cause is vitamin B12 deficiency [4,6]. The frequent association of macrocytic anemia, hyperpigmentation, low vitamin B12 levels in serum, the presence of low vitamin B12 levels in the mother, a vegetarian diet, and lack of proper complementary feeding favor this hypothesis. Moreover, improvement in the symptoms with vitamin B12 administration further corroborates the causal relationship [4,5,6].

In their study, Gupta et al. observed that in the absence of definite etiology, vitamin B12 supplementation along with iron, magnesium, and calcium constitute the mainstay of treatment. Some cases require additional medicines such as propranolol, phenobarbitone, and carbamazepine for controlling tremors [7]. In their review, Goraya et al. noted that treatment with vitamin B12 alone resulted in remarkable recovery in hematological and neurological symptoms [4]. Even administering a single intramuscular dose of 50 µg vitamin B12 to mothers of breastfed infants suffering from infantile tremor syndrome was found to be effective. Similar results were reported by Saraswat et al. and Gautam et al. [8,9]. In all studies, besides vitamin B12 administration, management of coexisting protein energy malnutrition and infections and counseling of the parents regarding proper weaning practice were done.

Most studies confirm that infantile tremor syndrome) is a self-limiting condition, usually resolving in four to six weeks [3,7]. Tremors usually resolve within one to two weeks [6). The disposition of the child turns cheerful from apathetic in about one to two weeks [4]. Skin and hair changes are slower to recover taking months [6]. The cognitive improvement and regaining developmental milestones occur over months to years [3,5]. However, a lower intelligence quotient (IQ) was noted in some cases at the end of 10 years [4,7]. Cerebral atrophy noted in MRI also takes months to resolve [8]. Hence, for assessment of IQ and cognitive deficits, long-term follow-up is required.

## Conclusions

Infantile tremor syndrome should be considered in a child presenting with developmental delay, abnormal movement, pallor, and skin and hair changes. It has long-term consequences on the development of the child. Hence, it is crucial to recognize it at the earliest to prevent long-term consequences on development and to treat it with vitamin B12 administration. Its existence and favorable response to treatment suggest that basic interventions such as the promotion of proper nutrition and timely complementary and weaning practices are needed for decreasing the incidence of this disease.
